# Interaction Between Phosphorus and Zinc on the Biomass Yield and Yield Attributes of the Medicinal Plant Stevia (*Stevia rebaudiana*)

**DOI:** 10.1100/tsw.2005.49

**Published:** 2005-05-10

**Authors:** Kuntal Das, Raman Dang, T. N. Shivananda, Pintu Sur

**Affiliations:** ^1^ Al-Ameen College of Pharmacy, Hosur Road, Bangalore 560027, Karnataka, India; ^2^ Indian Institute of Horticultural Research, Hessaraghatta, Bangalore, Karnataka, India; ^3^ Department of Agricultural Chemistry and Soil Science, Bidhan Chandra Krishi Viswavidyalaya, Mohanpur 741252, Nadia, West Bengal, India

**Keywords:** interaction, phosphorus, stevia, yield, zinc

## Abstract

A greenhouse experiment was conducted at the Indian Institute of Horticultural Research (IIHR), Bangalore to study the interaction effect between phosphorus (P) and zinc (Zn) on the yield and yield attributes of the medicinal plant stevia. The results show that the yield and yield attributes have been found to be significantly affected by different treatments. The total yield in terms of biomass production has been increased significantly with the application of Zn and P in different combinations and methods, being highest (23.34 g fresh biomass) in the treatment where Zn was applied as both soil (10 kg ZnSO_4_/ha) and foliar spray (0.2% ZnSO_4_). The results also envisaged that the different yield attributes viz. height, total number of branches, and number of leaves per plant have been found to be varied with treatments, being highest in the treatment where Zn was applied as both soil and foliar spray without the application of P. The results further indicated that the yield and yield attributes of stevia have been found to be decreased in the treatment where Zn was applied as both soil and foliar spray along with P suggesting an antagonistic effect between Zn and P.

## INTRODUCTION

The international business in medicinal and aromatic plants (MAPs)–based products has increased exponentially in recent years. It is estimated that about 80% of the population in developing countries rely on traditional medicine for primary health care. Most of the traditional drugs are derived from plant sources. Even in the modern pharmaceutical trade, the top-20, best-selling drugs have one or more constituents of plant origin. The global market size and business of medicinal plant materials including *Stevia rebaudiana* and health-care products based on these herbs comes to around 62 billion US$ and is likely to cross the 1 trillion mark by 2020 and 5 trillion by 2050[1].

Stevia (*S. rebaudiana*) is a natural, noncaloric, sweet herb originating from Paraguay. It has been an effective alternative to sugar for over 1500 years. Whitaker[2] reported that stevia is a sugar supplement that has been approved by the U.S. Food and Drug Administration (FDA) and can be grown as easily as any other vegetable, either a few in the house or a few hundred in the garden. The plant reaches a height of 45 cm in 3 months. Stevia has tremendous national as well as international demand. It has been selected as an alternative crop for West Bengal and India with high return support to the farmers[3]. Therefore, the cultivation of *S. rebaudiana* needs special attention with modern agrotechniques including balanced use of major, secondary, and micronutrient fertilizers. Stevia is grown in well-drained soil, such as red and sandy loam soil, with acidic reaction. The biomass yield of stevia can be increased with the balanced application of NP and K fertilizers, but the role of micronutrients (especially Zn) cannot be ignored, which might have favourable effect on the biomass production as well as quality of stevia. Such favourable effects of Zn on the biomass production, as well as yield attributes, are still lacking and hence the present investigation was undertaken.

## MATERIALS AND METHODS

Cuttings of the stevia plant were collected from Gandhi Krishi Vigyan Kendra, Bangalore and were used as a test plant. Before planting, initial soil samples were analysed for pH (Soil:Water, 1:2.5); organic carbon, available phosphorus, CEC, and DTPA-Zn by following the method as described by Jackson[4] and Lindsay and Norvell[5], respectively. After extracting the soil samples, Zn and P were determined with the help of an atomic absorption spectrophotometer (Perkin Elmer model AAnalyst 100) and spectrophotometer, respectively. The relevant physicochemical properties of soils were: pH, 8.9; organic carbon, 3.8 g/kg; available $P_2O_5$, 38 kg/ha; CEC, 14.4 Cmol($p^+$)/kg; DTPA-extractable Zn, 0.42 mg/kg.

Thirty-two numbers of earthen pots having 15-kg soil capacity were taken and 10-kg powdered soil collected from the IIHR farm (Hessaraghata, Bangalore) was filled up to each pot and the following treatments were: $T_1$: absolute control, no application of Zn and P; $T_2$: application of $P_2O_5$, but no application of Zn; $T_3$: soil application of Zn as $ZnSO_4$ @ 10 kg/ha, but no application of $P_2O_5$; $T_4$: foliar application of Zn as $ZnSO_4$ @ 0.2% solution, but no application of $P_2O_5$; $T_5$: both soil ($ZnSO_4$ @ 10 kg/ha) and foliar ($ZnSO_4$ @ 0.2%) of Zn, but no application of P; $T_6$: soil application of both Zn as $ZnSO_4$ @ 10 kg/ha and $P_2O_5$ as SSP @ 30 kg/ha; $T_7$: foliar application of Zn as $ZnSO_4$ @ 0.2% along with soil application of $P_2O_5$ at 30 kg/ha; $T_8$: both soil (Zn as $ZnSO_4$ @ 10 kg/ha) and foliar (Zn as $ZnSO_4$ @ 0.2%) application of Zn along with basal application of $P_2O_5$ as SSP @ 30 kg/ha. Each treatment was replicated four times in a completely randomised design (CRD). There were 32 pots altogether ($8 \times 4$). The pots were placed in net house for monitoring growth of the plant after putting cuttings of the stevia plant in each pot. Then the plants were allowed to grow for a period of 60 days. The periodic collection of soil and plant samples was made and analysed for pH, DTPA-extractable Zn, and available P by following the method as mentioned earlier. Besides, different yield attributes and yields were also recorded periodically.

## RESULTS AND DISCUSSION

### Interaction Effect Between Zinc and Phosphorus on the Yield and Yield Attributes of Stevia

### *Biomass Yield*

The results (Table 1) show that the amount of fresh biomass yield has been found to increase progressively irrespective of treatments. However, the magnitude of such increase varied with treatments, being recorded highest (6.99 g) in treatment $T_5$ at 30 days of plant growth, which was closely followed by 6.81 g in treatment $T_4$. The total fresh biomass production was also recorded highest (23.34 g) after 60

**TABLE 1**
**Interaction Effect Between Zn and P on the Fresh Biomass Yield (g) of Stevia Plant (*S. rebaudiana*) (Mean of Four Replications)**

| Treatments | 15 Days | 30 Days | 45 Days | 60 Days | Total Biomass (g) |
|---|---|---|---|---|---|
| –Zn, –P ($T_1$) | 1.84 | 6.27 | 5.73 | 5.38 | 21.61 |
| –Zn, +P ($T_2$) | 2.73 | 5.11 | 5.88 | 4.68 | 20.26 |
| Zn(S), –P ($T_3$) | 1.98 | 6.68 | 6.10 | 6.38 | 22.52 |
| Zn(F), –P ($T_4$) | 1.90 | 6.81 | 5.11 | 5.35 | 21.11 |
| Zn(S+F), –P ($T_5$) | 2.91 | 6.99 | 5.32 | 6.50 | 23.34 |
| Zn(S), +P ($T_6$) | 2.73 | 6.07 | 5.88 | 6.28 | 22.54 |
| Zn(F), +P ($T_7$) | 1.76 | 5.01 | 6.11 | 4.92 | 19.25 |
| Zn(S+F), +P ($T_8$) | 1.74 | 4.98 | 5.22 | 3.94 | 17.48 |
| CD ($p$ = 0.05) | 0.16 | 0.23 | 0.19 | 0.11 | 0.21 |

S = soil application; F = foliar application.

days of plant growth in treatment $T_5$, which might be due to application of Zn both as soil and foliar spray only and no application of P. However, the lowest fresh biomass yield (1.74 g) was recorded in treatment $T_8$ at 15 days of plant growth and the total biomass was also recorded lowest (17.48 g) in treatment $T_8$ where Zn was applied as both soil and foliar spray in the presence of P, which might be due to an antagonistic effect between them. Chalapathi et al.[6,7] reported that the application of NP and K at 60, 30, and 45 kg/ha, respectively, produced higher dry leaf yield with the simultaneous higher nutrient uptake by stevia plant. Sood and Kumar[8] also reported that green and dry foliage yield increased with increasing levels of N and P which also confirmed the results obtained in the present investigation.

## *Yield Attributes*

The results (Table 2) show that the mean height of the stevia plant gradually increases with the progress of growth up to 60 days irrespective of treatments. However, the changes in height were varied with treatments, being highest (36 cm) in treatment $T_5$ at 60 days of growth where Zn was applied as both soil and foliar spray in the absence of P, while that of the same value significantly decreased (34 cm) with the combined application of Zn and P either as soil or foliar spray. Such decrease in the mean height due to their combined application might be explained by the antagonistic effect between Zn and P.

The results (Table 3) show that the total number of branches per pot gradually increases with the progress of plant growth with maximum branches (29/pot) at 60 days after plant growth in treatment $T_5$ where Zn was applied as both soil and foliar spray in the absence of P, while that of the same value significantly decreased (23/pot) at 60 days of growth due to combined application of Zn and P suggesting an antagonistic effect between them.

The results (Table 4) show that an average number of leaves per plant also followed an increasing trend with the progress of plant growth with maximum value at 45 days of growth and thereafter the amount of the same decreases up to 60 days of growth irrespective of treatments. The average number of leaves per plant has been recorded highest (59.65) in treatment $T_5$ where Zn was applied as both soil and foliar spray in the absence of P at 60 days of plant growth. The results also showed that an average number of leaves per plant has been found to be significantly increased with both soil and foliar applications of only Zn in the absence of P as compared to the treatments receiving combined applications of Zn and P either as soil or foliar spray, which might be due to the antagonistic effect between them as

**TABLE 2**
**Interaction Effect Between Zn and P on the Height of Plant (cm) of Stevia (*S. rebaudiana*) (Mean of Four Replications)**

| Treatments | Height/Plant (cm) | | | |
|---|---|---|---|---|
| | 15 Days | 30 Days | 45 Days | 60 Days |
| –Zn, –P ($T_1$) | 11.00 | 23.00 | 32.00 | 34.00 |
| –Zn, +P ($T_2$) | 11.00 | 22.00 | 32.00 | 35.00 |
| Zn(S), –P ($T_3$) | 12.00 | 22.00 | 31.00 | 35.00 |
| Zn(F), –P ($T_4$) | 12.00 | 24.00 | 32.00 | 35.00 |
| Zn(S+F), –P ($T_5$) | 13.00 | 24.00 | 33.00 | 36.00 |
| Zn(S), +P ($T_6$) | 11.00 | 22.00 | 31.00 | 34.00 |
| Zn(F), +P ($T_7$) | 12.00 | 23.00 | 32.00 | 34.00 |
| Zn(S+F), +P ($T_8$) | 11.00 | 22.00 | 32.00 | 35.00 |
| CD ($p$ = 0.05) | 0.27 | 0.23 | 0.31 | 0.43 |

S = soil application; F = foliar application.

**TABLE 3**
**Interaction Effect Between Zn and P on the Total Number of Branches of Stevia (*S. rebaudiana*) (Mean of Four Replications)**

| Treatments | Total Number of Branches | | | |
|---|---|---|---|---|
| | 15 Days | 30 Days | 45 Days | 60 Days |
| –Zn, –P ($T_1$) | 12 | 19 | 22 | 22 |
| –Zn, +P ($T_2$) | 15 | 18 | 24 | 25 |
| Zn(S), –P ($T_3$) | 12 | 19 | 27 | 27 |
| Zn(F), –P ($T_4$) | 14 | 23 | 29 | 29 |
| Zn(S+F), –P ($T_5$) | 8 | 13 | 26 | 29 |
| Zn(S), +P ($T_6$) | 16 | 17 | 23 | 25 |
| Zn(F), +P ($T_7$) | 11 | 18 | 28 | 26 |
| Zn(S+F), +P ($T_8$) | 9 | 18 | 22 | 23 |
| CD ($p$ = 0.05) | 2.10 | 1.50 | 1.38 | 1.46 |

S = soil application; F = foliar application.

reported by Mandal and Haldar[9]. The results clearly indicated that the number of leaves per plant was found independent with the increased number of branches, which might be due to relatively more accumulation of Zn compared to P resulting from their strong antagonistic effect, causing more leaf production.

## CONCLUSIONS

The interaction effect between Zn and P did not show any positive effect on the biomass yield and yield attributes of stevia (*S. rebaudiana*) plant. The results clearly suggested that the application of only Zn as both soil and foliar spray was found superior over that of only P as basal application in relation to increase in biomass yield and yield attributes of the medicinal plant stevia.

**TABLE 4**
**Interaction Effect Between Zn and P on the Number of Leaves per Plant of Stevia (*S. rebaudiana*) (Mean of Four Replications)**

| Treatments | Number of Leaves per Plant | | | |
|---|---|---|---|---|
| | 15 Days | 30 Days | 45 Days | 60 Days |
| –Zn, –P ($T_1$) | 24.25 | 51.25 | 59.25 | 51.25 |
| –Zn, +P ($T_2$) | 25.25 | 48.50 | 61.00 | 54.25 |
| Zn(S), –P ($T_3$) | 31.75 | 49.75 | 61.50 | 57.75 |
| Zn(F), –P ($T_4$) | 28.50 | 60.00 | 62.75 | 48.00 |
| Zn(S+F), –P ($T_5$) | 29.50 | 60.50 | 63.75 | 59.65 |
| Zn(S), +P ($T_6$) | 27.00 | 52.75 | 53.25 | 35.50 |
| Zn(F), +P ($T_7$) | 26.25 | 46.00 | 56.25 | 38.75 |
| Zn(S+F), +P ($T_8$) | 24.25 | 45.25 | 54.50 | 33.25 |
| CD ($p$ = 0.05) | 0.81 | 0.32 | 0.24 | 0.74 |

S = soil application; F = foliar application.

## ACKNOWLEDGMENTS

The authors are grateful to the Department of Agricultural Chemistry and Soil Science, Bidhan Chandra Krishi Viswavidyalaya, Mohanpur, Nadia, West Bengal for rendering help and cooperation in analysing soil and plant samples with the help of an atomic absorption spectrophotometer (Perkin Elmer Model AAnalyst 100) during the course of the present investigation as a part of postgraduate dissertation work.

## BIOSKETCHES

**Kuntal Das**, lecturer in pharmacognosy in St. John's Pharmacy College, Bangalore, India. Sri Das has published five papers relating to medicinal and aromatic plants with particular reference to Stevia. He received second prize for a poster presentation at the International Convention of APTI (Association of Pharmaceutical Teachers of India) held in Visakhapatnam, 2-3th October, India. 2004.

**Raman Dang** is an Assistant Professor in the Department of Pharmacognosy, Al-Ameen College of Pharmacy, Hosur Road, Bangalore, Karnataka, India. Dr. Dang has published some good-quality research papers on medicinal and aromatic plants with special reference to pharmaceutical research. He received the Best Oral Presentation Award at the International Conference on Promotion of Botanicals at Jadavpur, Kolkata, India held February, 2005.

**T.N. Shivananda** is a senior scientist in the Division of Medicinal and Aromatic Plants, Indian Institute of Horticultural Research, Hessaraghatta, Bangalore, Karnataka, India. Dr. Shivananda has published a good number of original research papers in journals of national and international repute.

**Pintu Sur** is a Ph.D. student in the Department of Agricultural Chemistry and Soil Science, Bidhan Chandra Krishi Viswavidyalaya, Mohanpur, Nadia, West Bengal, India whose field of specialization is soil chemistry, soil fertility, and plant nutrition. Sri Sur has published three research papers relating to soil chemistry and plant nutrition with particular reference to micronutrients.
